# Alzheimer's Disease Classification With a Cascade Neural Network

**DOI:** 10.3389/fpubh.2020.584387

**Published:** 2020-11-03

**Authors:** Zeng You, Runhao Zeng, Xiaoyong Lan, Huixia Ren, Zhiyang You, Xue Shi, Shipeng Zhao, Yi Guo, Xin Jiang, Xiping Hu

**Affiliations:** ^1^Department of Neurology, Shenzhen People's Hospital, The First Affiliated Hospital of Southern University of Science and Technology, The Second Clinical Medical College of Jinan University, Shenzhen, China; ^2^Shenzhen Institutes of Advanced Technology, Chinese Academy of Sciences, Shenzhen, China; ^3^The First Affiliated Hospital, Jinan University, Guangzhou, China; ^4^Department of Geriatrics, Shenzhen People's Hospital, The First Affiliated Hospital of Southern University of Science and Technology, The Second Clinical Medical College of Jinan University, Shenzhen, China

**Keywords:** Alzheimer's disease, deep learning, automatic diagnosis, gait, EEG

## Abstract

Classification of Alzheimer's Disease (AD) has been becoming a hot issue along with the rapidly increasing number of patients. This task remains tremendously challenging due to the limited data and the difficulties in detecting mild cognitive impairment (MCI). Existing methods use gait [or EEG (electroencephalogram)] data only to tackle this task. Although the gait data acquisition procedure is cheap and simple, the methods relying on gait data often fail to detect the slight difference between MCI and AD. The methods that use EEG data can detect the difference more precisely, but collecting EEG data from both HC (health controls) and patients is very time-consuming. More critically, these methods often convert EEG records into the frequency domain and thus inevitably lose the spatial and temporal information, which is essential to capture the connectivity and synchronization among different brain regions. This paper proposes a cascade neural network with two steps to achieve a faster and more accurate AD classification by exploiting gait and EEG data simultaneously. In the first step, we propose attention-based spatial temporal graph convolutional networks to extract the features from the skeleton sequences (i.e., gait) captured by Kinect (a commonly used sensor) to distinguish between HC and patients. In the second step, we propose spatial temporal convolutional networks to fully exploit the spatial and temporal information of EEG data and classify the patients into MCI or AD eventually. We collect gait and EEG data from 35 cognitively health controls, 35 MCI, and 17 AD patients to evaluate our proposed method. Experimental results show that our method significantly outperforms other AD diagnosis methods (91.07 vs. 68.18%) in the three-way AD classification task (HC, MCI, and AD). Moreover, we empirically found that the lower body and right upper limb are more important for the early diagnosis of AD than other body parts. We believe this interesting finding can be helpful for clinical researches.

## 1. Introduction

Alzheimer's disease (AD) is the most common cause of cognitive impairment and is one of the diseases with the highest incidence among the elderly. In 2006, 26.6 million people on the earth suffered from AD, and the number is still rapidly increasing every year (1). More critically, AD has become the seventh leading cause of death (2). Conventional AD diagnosis methods often use scale screening and brain imaging equipment such as functional Magnetic Resonance Imaging (fMRI), Computer Tomography (CT), and Positron Emission Tomography (PET). These methods require experienced clinicians as well as exhaustive examinations.

Recently, many studies (3–9) have been conducted to reduce the diagnosis cost and shorten the diagnosis time by designing an AD classification system that is able to detect and classify AD automatically. However, it is challenging to classify AD precisely for the following reasons: on the one hand, the prodromal stage of AD, namely mild cognitive impairment (MCI), has a light symptom, making it hard to detect; On the other hand, extracting robust features for AD detection is very challenging due to the limited volume of medical data.

Previous studies on AD classification exploit gait data (3, 10–17) due to the strong relationship between gait features and cognitive function (18–25). They often extract hand-crafted features from the input gait data (e.g., skeleton) and classify AD relying on these features. However, designing hand-crafted features for AD classification requires expert knowledge, and it is difficult to generalize the hand-crafted features to other tasks. Recently, some researchers (12, 13, 15, 16, 26, 27) attempt to conduct AD classification using EEG data. However, existing EEG-based methods often (6, 7) need to convert EEG data into frequency domain information and calculate the Power Spectral Density (PSD) features for classification. In this sense, these methods will inevitably lose the information in the spatial and time domains of EEG data, which, however, is very important for capturing the coherence and synchronizations among different brain regions. It is worth noting that existing methods use one modal only (gait or EEG data) and suffer from the following limitations: (1) as discussed in (28, 29), using gait data can accurately distinguish HC and patients but often fails to classify MCI and AD, and (2) using EEG data can classify MCI and AD more accurately, but it is time-consuming to collect EEG data from both HC and patients.

We contend that considering the two modalities (i.e., gait and EEG data) simultaneously helps achieving faster and more accurate classification. To this end, we propose a cascade neural network with two steps for the early diagnosis of AD using both gait data and EEG data simultaneously. **In the first step**, we use gait data to classify HC and patients. For the purpose of reducing the psychological disturbance to the subject, we follow (10) to use the Kinect devices as the acquisition equipment to capture skeleton sequences. Regrading the non-Euclidean skeleton data, we propose to use attention-based spatial temporal graph convolutional networks (AST-GCN) to model the relationships among body key points and automatically extract powerful features for distinguishing between HC and patients. **In the second step**, we use the original EEG data to distinguish MCI and AD patients further. Unlike other methods that convert EEG data to the frequency domain, we propose spatial temporal convolutional networks (ST-CNN) to directly extract the spatial and temporal features from original EEG data and use them to classify MCI and AD. In this manner, the EEG data from HC are no longer required, saving a lot of data collection time. We collect a data set consisting of gait and EEG data from 35 cognitively health controls, 35 MCI patients, and 17 AD patients to evaluate our proposed method.

Our main contributions are summarized as follows:

- We propose a cascade neural network that uses both gait and EEG data to classify AD, which achieves a high accuracy rate with less manual participation. This is the first attempt to consider two modalities for AD classification to the best of our knowledge.
- We propose attention-based spatial temporal graph convolutional networks to automatically extract the features from gait data and leverage them to classify AD.
- Moreover, we also propose spatial temporal convolutional networks to fully extract the spatial and temporal features from the original EEG data in both space and time domains.
- The accuracy rate of our proposed cascade neural network in the three-way classification of HC, MCI, and AD reaches 91.07%, which is much higher than the method using one modal (68.18%). The accuracy of HC vs. MCI/AD is up to 93.09%.

The rest of the paper is arranged as follows: Related work is concentrated on section 2; Section 3 details the proposed framework and the modules in it; Experimental results are exhibited in section 4; Section 5 concludes this paper.

## 2. Related Work

Gait data has been used extensively to classify AD. Wang et al. (3) developed a device to collect the inertial signals of subjects. They designed an algorithm to leverage the inertial signals to detect and calculated the features of the stride. Then they selected the salient features to classify HC and AD. The classification accuracy rates in the female and the male groups are 70.00 and 63.33%, respectively. Choi et al. (29) compared the gait and cognitive function between the HC group and MCI/AD groups. They found that gait features can distinguish MCI and HC, while cognitive tests are suitable for distinguishing AD and HC. The average detection rate of AD and MCI from HC using gait variables is 75%. Seifallahi et al. (10) used Kinect to collect gait data, extracted, and screened the features. Then they used Support Vector Machines (SVM) to classify AD and HC. The classification accuracy rate is 92.31%. Varatharajan et al. (4) used IoT devices to collect gait data and then extracted the features using the dynamic time warping (DTW) algorithm. The accuracy rate of classification is about 70%. Although the above works achieve good performance, they all rely on handcrafted feature extraction, which cannot guarantee the full use of the implicit information in gait data, and the features designed for specific tasks cannot be applied to other general tasks. The attention-based spatial temporal graph convolutional networks we proposed can automatically extract gait data features and exploit the relationships among body joints.

EEG data is another important information that can be used to diagnose AD. Existing methods for the early diagnosis of AD using EEG data can be categorized into **handcrafted feature based-methods** and **deep learning methods**. Anderer et al. (12) and Pritchard et al. (13) input EEG markers into an ANN to perform a binary classification between AD and HC with an accuracy rate of 90%. Trambaiolli et al. (15) extracted features based on coherence and used Support Vector Machines(SVM) to classify AD and HC, with 79.9% accuracy. Rossini et al. (16) tested the IFAST procedure to classify HC and MCI, achieving 93.46% accuracy. These methods all require handcrafted feature extraction. In recent years, more and more deep learning methods have been applied to the classification of AD. Ieracitano et al. (6) calculated the PSD features of the subject's EEG data. They converted the PSD features into images, and then used the convolutional neural networks for the early diagnosis of AD, achieving an accuracy of 89.8% in the binary classification and 83.3% in three-way classification. Bi and Wang (7) calculated the PSD features of EEG data, then used the feature representation method proposed by (30) to convert the PSD features into images. They designed a DCssCDBM with a multi-task learning framework, achieving an accuracy of up to 95.05%. These deep learning methods all need to convert EEG data into frequency domain information. This way will lose the information in the spatial and temporal domains of EEG data, which is essential for capturing coherence and synchronization among different brain regions. We directly use the original EEG data containing both spatial and temporal information. We propose spatial temporal convolutional networks to extract the temporal and spatial implicit features of EEG data.

The methods mentioned above leveraged either gait data or EEG data only for the early diagnosis of AD. The gait data collection procedure is simple, short in time, and easy to operate, but there is no significant difference in gait features between MCI and AD (29), and thus method relying on gait data cannot classify AD and MCI precisely. Conversely, EEG data can provide promising cues to classify AD and MCI, but the acquisition process is complicated and takes a long time. We consider gait and EEG data simultaneously to achieve a fast and accurate classification of AD.

## 3. Proposed Method

**Notation**. Let $S={\left\{s_{i}\right\}}_{i=1}^{N_{s}}$ be the subject set that includes *N*_*s*_ subjects, where *s*_*i*_ represents the *i*^*th*^ subject. Let $G_{i}={\left\{g_{i}^{j}\right\}}_{j=1}^{N_{g}}$ denote clip set where *N*_*g*_ clips are sampled from the gait data of the *i*^*th*^ subject *s*_*i*_, where $g_{i}^{j}$ represents the *j*^*th*^ clip. Let $E_{i}={\left\{\varepsilon_{i}^{e}\right\}}_{e=1}^{N_{e}}$ denote the epoch set containing *N*_*e*_ epochs sampled from the EEG data of the *i*^*th*^ subject *s*_*i*_, where $\varepsilon_{i}^{e}$ represents the *e*^*th*^ epoch.

**Problem Definition**. Given gait clip set $G_{i}$ and EEG epoch set $E_{i}$ of subject *s*_*i*_, the classification of AD aims to map physiological signals, $G_{i}$ and $E_{i}$, into HC, MCI, and AD groups corresponding to the state of subject *s*_*i*_. This task is very challenging due to the limited volume of data and the subtle differences among the three groups, especially for HC and MCI.

### 3.1. Pipeline Overview

Existing methods used either gait data or EEG data only for the classification of AD. However, as discussed in (28, 29), using gait data can accurately distinguish HC and patients, but the methods using gait data only often fail to classify MCI and AD. For the EEG data that are more sensitive to the differences between MCI and AD, some studies used EEG data to classify AD. However, collecting EEG data from both HC and patients takes a lone time. We believe that combining the two is able to make the early diagnosis of AD faster and more accurately. This drives us to propose a cascade neural network for the early diagnosis of AD with both gait and EEG data.

**Figure 1:**
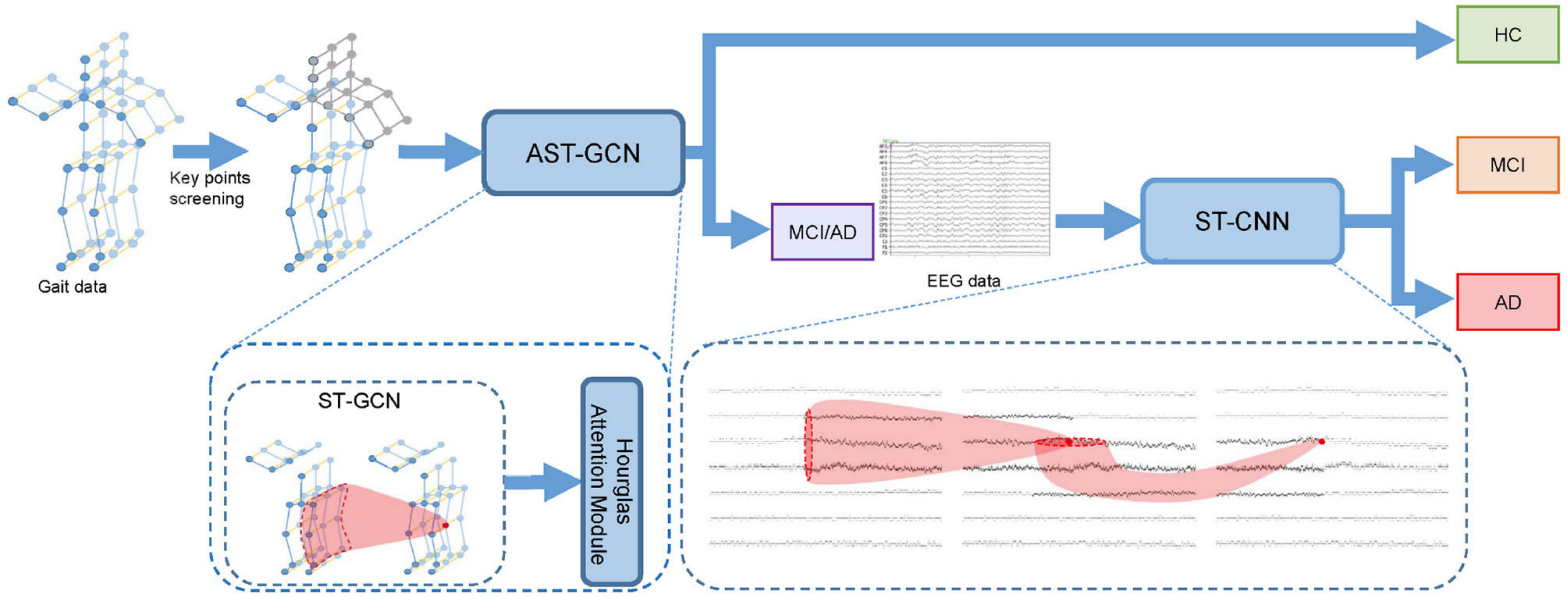
Cascade neural network for the early diagnosis of AD. We perform key point screening on gait data to form key-point skeleton sequences. Then we use attention-based spatial temporal graph convolutional networks (AST-GCN) to extract features and classify the subject into HC or MCI/AD with features. If the subject is classified into MCI/AD, we will input the EEG data into spatial temporal convolutional networks (ST-CNN) to extract features and perform MCI vs. AD binary classification.

Given gait clip $g_{i}^{j}$ and EEG epoch $\varepsilon_{i}^{e}$ of subject *s*_*i*_, we conduct the classification in two steps. Firstly, we use gait data to distinguish HC and MCI/AD patients. In this step, we select key points from $g_{i}^{j}$ to form key-point skeleton sequences first. Then we input the key-point skeleton sequences into attention-based spatial temporal graph convolutional networks (AST-GCN) to extract features. Finally, we use these features to classify HC and MCI/AD by a standard SoftMax classifier. We further distinguish AD from MCI with EEG epoch $\varepsilon_{i}^{e}$ in the second step. We input $\varepsilon_{i}^{e}$ into the spatial temporal convolutional networks (ST-CNN) to extract the implicit features in spatial and temporal domain. We then used the features extracted by ST-CNN for the binary classification of MCI vs. AD. In our method, the EEG data from HC are not required. The architecture of our proposed framework is shown in Figure 1.

### 3.2. Attention-Based Spatial Temporal Graph Convolutional Networks

Existing methods that use gait data for the early diagnosis of AD rely on handcrafted features, which are inefficient and cannot fully use implicit information in gait data. We need to automatically extract the implicit features in gait data for the early diagnosis of AD, which is the strength of deep learning. Our gait data is composed of skeleton sequences recognized by the Kinect devices. Traditional deep learning methods such as convolutional networks cannot handle such non-Euclidean data. The ST-GCN proposed by (31) shows an excellent performance in extracting the features from skeleton sequences. We apply it as our basic model to the classification of AD and propose attention-based spatial temporal graph convolutional networks (AST-GCN) according to our task and data characteristics. Based on clinical experience and experimental comparison results, we found that different body parts have different importance in the classification of AD. For this reason, given skeleton sequences, we first perform key point filtering to form our key-point skeleton sequences and then input it into the proposed attention-based spatial temporal graph convolution networks. The extracted spatial and temporal features are finally used for classification. In the next few subsections, we will first briefly introduce ST-GCN, then we will introduce how we do key point filtering and the proposed attention-based spatial temporal graph convolutional networks.

#### 3.2.1. Spatial Temporal Graph Convolutional Networks

**Figure 2:**
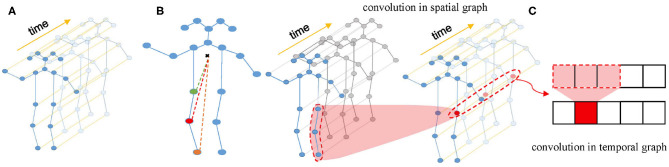
**(A)** Spatial temporal graph of skeleton sequences. **(B)** The “Spatial Configuration” strategy. **(C)** The architecture of ST-GCN.

Firstly, a spatial temporal graph is constructed from skeleton sequences, as shown in Figure 2A. The edges of the spatial temporal graph consist of two parts. One part is the natural connections between joint points of the human skeleton in a single frame called spatial edges, and the other part is the time edges formed by connecting the same joint points between adjacent frames. Then, the input features composed of the coordinate vectors of the nodes in the graph are inputted into multiple layers of spatial-temporal graph convolution. Defining the weight function of the graph convolution operation can be realized by a variety of strategies for partitioning each node's neighborhood point set. Experiments show that the “Spatial Configuration” strategy, as shown in Figure 2B, works best. According to this strategy, the neighborhood point set of the root node (red node) is divided into three subsets, namely: (1) The root node itself (red node); (2) The centripetal group (orange node): the nodes closer to the gravity center of the skeleton than the root node; (3) centrifugal group (green node): the nodes that are farther from the gravity center of the skeleton than the root node. The formula of space graph convolution can be written as:

(1) $$\begin{array}{r} f_{out}=\Lambda^{-\frac{1}{2}}\left(\text{A}+\text{I}\right)\Lambda^{-\frac{1}{2}}f_{in}\text{W}, \end{array}$$

where *f*_*in*_ denotes the feature map of the clip composed of the coordinates of input skeleton sequences, which is a *D* × *T* × *V* matrix, where *D* = 3 corresponds to Three coordinates (*x, y, z*), *T* represents the time points i.e., the number of frames of the skeleton sequences, *V* is the number of nodes that constitute the spatial graph in each frame. **W** is the weight function; Λ is the degree matrix of the spatial graph; **A** is the adjacency matrix of the spatial graph; **I** is the self-connection matrix. Moreover, **M** is proposed as a learnable edge weight, which has the same size as the adjacency matrix. It is used in every layer of spatial temporal graph convolution. Then the Equation (1) can be written as:

(2) $$\begin{array}{r} f_{out}=\Lambda^{-\frac{1}{2}}\left(\left(\text{A}+\text{I}\right)⊙\text{M}\right)\Lambda^{-\frac{1}{2}}f_{in}\text{W}, \end{array}$$

where ⊙ notes the element-wise multiply. Spatial temporal convolution module consists of a convolution in the spatial graph and a convolution in the temporal graph. The structure of spatial temporal convolution module is shown in Figure 2C.

#### 3.2.2. Key Points Filtering

Several studies (18, 20–24, 32) found that the AD group has significant differences with the HC group in gait speed, gait cadence, stride et al. This means that the joints of the lower body, such as the ankles, are more critical for the early diagnosis of AD. Besides, Most subjects are right-handed. It is clinically believed that the left hemisphere of right-handed patients is more sensitive to AD and more likely to be affected. When we observe the learnable parameter **M** of the basic model after it converges, we find that the connections among the joint points of the lower body and the right upper limb are given higher weights, which means that these joint points are more important than other parts. Through experimental comparison, we also verified that performance classification with the skeleton sequences composed of the joint points of the lower body and the right upper limb are better than that with the skeleton sequences composed of other parts. Therefore, we select the joint points of the lower body and the right upper limb to form key-point skeleton sequences.

#### 3.2.3. Hourglass Attention Module

**Figure 3:**
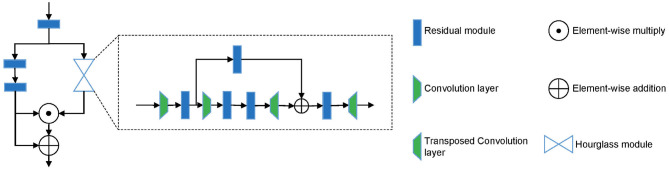
The structure of hourglass attention module.

From the description above, we can see that different parts are of different importance for the early diagnosis of AD. We argue that even in the key-point skeleton sequences we construct, joints in some parts are more important than other parts, such as ankles and wrists. Therefore, to drive the model further focus on important joints, we introduced an hourglass attention module with a structure similar to the attention module in (33). However, we replaced the pooling layer with a convolutional layer in the time domain with a stride of 4. The structure of the hourglass attention module is shown in Figure 3.

### 3.3. Spatial Temporal Convolutional Networks

Existing deep learning methods that use EEG data for the classification of AD convert EEG data into frequency domain information, then calculate PSD features and convert them into images. This way will lose the information in the time domain or even the spatial domain, which is essential to capture coherence and synchronization among different brain regions. The EEGnet proposed by (34) extracts the temporal and spatial features of original EEG data to recognize task-state EEG and shows good performance. However, its feature extraction in the spatial domain of EEG data simply uses a convolution layer to map the data to a single value. We believe that this is not able to fully extract the spatial features of EEG data. We propose the spatial temporal convolutional networks to extract features from original EEG data. Each ST-CNN module consists of a spatial convolution layer with a kernel size of *K*_*s*_ × 1 and a temporal convolution layer with a kernel size of 1 × *K*_*t*_ similar to (31). In this way, the EEG data is alternately convoluted in the space domain and the time domain through multiple ST-CNN layers to fully extract the implicit features in space and time. The structure of spatial temporal convolutional networks is shown in Table 1.

**Table 1:** The structure of spatial temporal convolutional networks, where *K*_*s*_ and *K*_*t*_ are the size of the kernel used in the spatial convolution layer and the temporal convolution layer in a ST-CNN module, respectively.

**Layer**	**Input channels**	**Operation**	**Kernel size**	**Stride**	**Output channels**
0	3	Batch normalization	–	–	3
1	3	ST-CNN	*K*_*s*_ = 1, *K*_*t*_ = 33	1	4
2	4	ST-CNN	*K*_*s*_ = 15, *K*_*t*_ = 33	4	4
3	4	ST-CNN	*K*_*s*_ = *C, K*_*t*_ = 33	1	16
4	16	ST-CNN	*K*_*s*_ = 1, *K*_*t*_ = 33	4	8
6	8	Flatten	–	–	*T*/2
Classifier	*T*/2	Full connection	–	–	*N*
	*N*	SoftMax	–	–	*N*

*C is the number of EEG channels. T is the number of time points. N is the number of classes. In the second layer, we use depthwise separable convolutions. In the 2nd and 4th ST-CNN module, we set stride to 4 as the pooling layer. The residual mechanism is used in each ST-CNN module*.

## 4. Experiments

### 4.1. Data Acquisition and Preprocessing

We collect gait data in cooperation with the Shenzhen Institute of Advanced Technology, Chinese Academy of Sciences and the Shenzhen People's Hospital, and the EEG data are collected by the Shenzhen People's Hospital. All MCI and AD patients are diagnosed by experienced neurologists based on the Montreal Cognitive Assessment(MoCA) and Mini-Mental State Examination (MMSE). We divide the subjects into three groups: HC, MCI, and AD. These groups include 35 cognitively healthy controls, 35 MCI patients, and 17 AD patients with mild-to-severe AD, respectively. The grouping criteria are shown in Table 2. We collect both gait and EEG data for MCI and AD patients, and only collect gait data for cognitively healthy controls.

**Table 2:** The grouping criteria for HC, MCI, AD.

	**HC**	**MCI**	**AD**
MoCA	> 30	18 ~ 30	0 ~ 17
MMSE	≥ 24	≥ 24	<24

#### 4.1.1. Gait Data

##### 4.1.1.1. Data Acquisition

**Figure 4:**
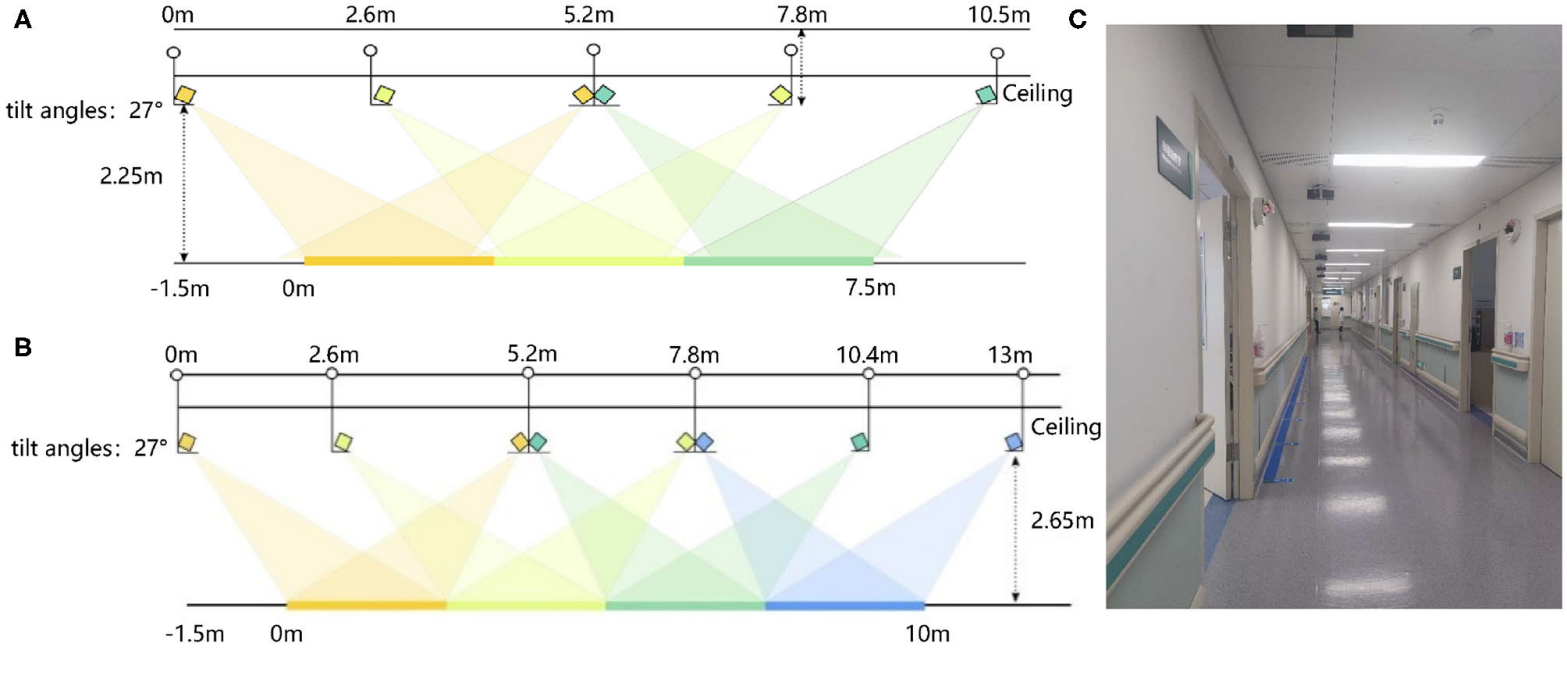
The deployment diagram of Kinect V2.0 devices: **(A)** The deployment diagram of devices in the Neurology Department. **(B)** The deployment diagram of devices in the Geriatrics Department. **(C)** The diagram of the actual data acquisition scene.

Gait data of 52 MCI and AD patients and 35 control subjects are collected in the Neurology and the Geriatrics Departments of Shenzhen People's Hospital, respectively. Our data collection settings are similar to (35). We use Microsoft Kinect V2.0 cameras as our data acquisition devices. The subjects are asked to walk at their natural and comfortable speed and posture under the devices. They walk a round trip on a straight path about 10 m. We deploy 8 and 6 devices in the Neurology and Geriatrics Department, respectively. The deployment diagram is shown in Figure 4. The tilt angle of all devices was set 27°.

##### 4.1.1.2. Data Preprocessing

**Figure 5:**
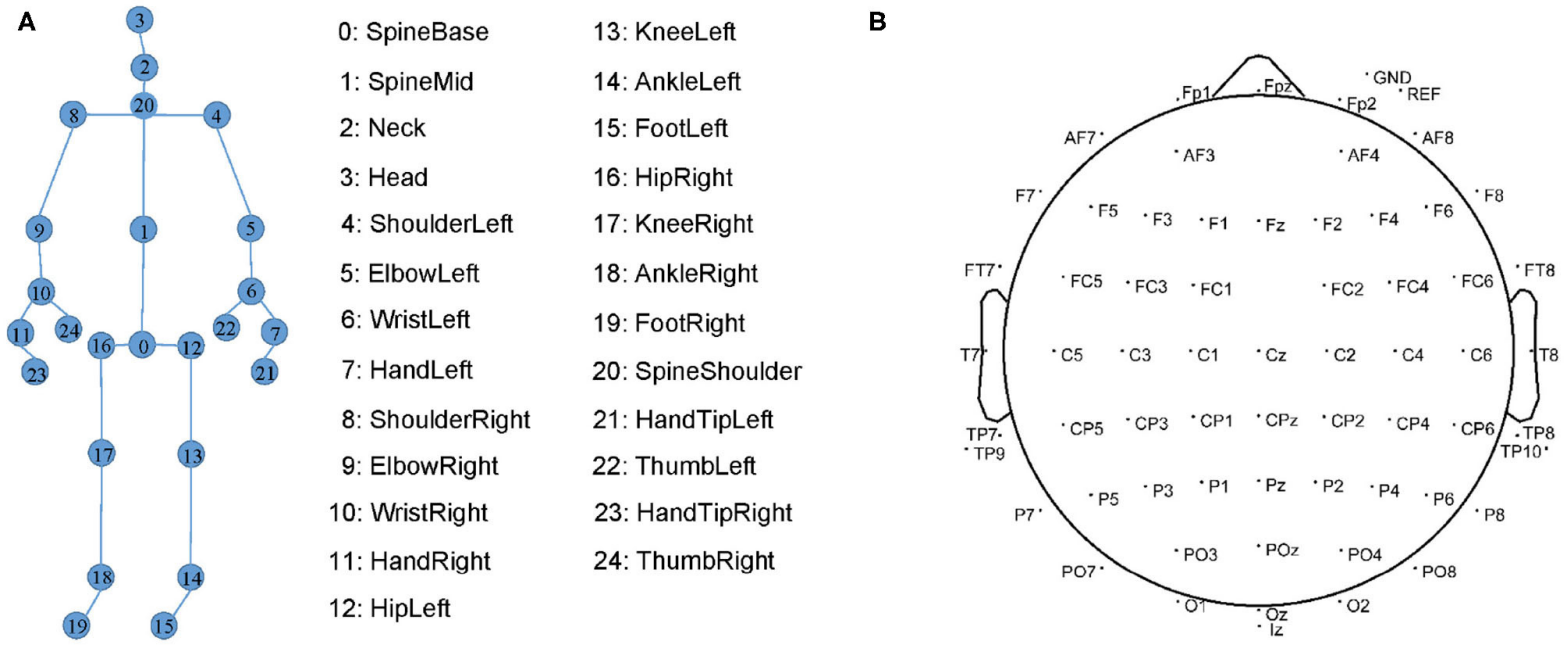
**(A)** The 25 markers on human skeleton recognized by Kinect. **(B)** 64 EEG electrode locations in the International 10-20 System.

Our gait data consists of the skeleton sequences recognized by the devices. Each skeleton is composed of three-dimensional coordinates of 25 joints. Their indexes are shown in Figure 5A. In each recording, the devices estimate the skeleton joint coordinates from both the front and back views. However, the skeletons estimated from the back view are less accurate than those from the front view. Therefore, we only select the skeletons from the front view as gait data.

Due to the venue restrictions, the data acquisition devices for patients and the devices for heath controls are deployed in different environments, which may cause differences in absolute coordinates of key points. To eliminate these differences, we follow (36) to perform the following coordinate transformation on the collected gait data in the data preprocessing stage. Since our devices are mounted on the ceiling, and there is an angle of 27° with the horizontal, we first rotate the coordinates [*x, y, z*] around the x-axis by −27° by calculating

(3) $$\left|\begin{array}{c} x\prime \\ y\prime \\ z\prime \end{array}\right|={\text{R}}_{\text{x}}\times \left|\begin{array}{c} x\\ y\\ z \end{array}\right|,\text{where }{\text{R}}_{\text{x}}=\left|\begin{array}{ccc} 1 & 0 & 0\\ 0 & cos\theta & -sin\theta \\ 0 & sin\theta & cos\theta \end{array}\right|,\;\theta =-27^{{}^{\circ}}.$$

In this way, the skeleton sequences are in a horizontal position relative to the cameras. We then move the origin of the coordinates to the base of the human spine, namely point 0, by computing

(4) $$\begin{array}{r} v_{\tau p}^{\prime }={\text{v}}_{\tau p}-{\text{v}}_{\tau 0}, \end{array}$$

where v_τ*p*_ is a coordinate vector of *pth* joint point of the skeleton in τ*th* frame. Moreover, the time lengths of gait records are different. Similar to (37), we intercept several clips of data from each gait record through a sliding window to make the number of clip frames consistent. We set the sliding window with a size of 60 frames and a stride of three frames. In this way, we have a total of 5,519 clips, and each gait clip $g_{i}^{j}$ is a matrix with a dimension of *D* × *T* × *V*, where *D* = 3, *T* = 60, *V* = 25.

#### 4.1.2. EEG Data

##### 4.1.2.1. Data acquisition

The EEG data are collected by the Neurology Department, Shenzhen People's Hospital. Due to a large mount of artifacts (e.g., myoelectricity) during human walking, the collected EEG data are in low quality. We follow (6, 38) to collect higher-quality resting EEG data. We collect the EEG data of the patients with eyes closed and with eyes open for 8 min each. We place 64-channel EEG electrodes on the patient's scalp at the standard locations during data acquisition as shown in Figure 5B. The EEG signals are recorded at a sampling frequency of 5,000 Hz.

##### 4.1.2.2. Data preprocessing

After EEG records are collected, we first remove artifacts from EEG records, such as electrooculograms and myoelectricity. Then we re-reference the data. The EEG signals of the Ref and Gnd electrodes are removed, and the average value of the remaining 62 channels is used as a reference value to recalculate the value of the EEG data. Using the original EEG data with a sampling rate of 5,000 Hz in our ST-CNN will inevitably incur large computation cost. Specifically, the input size is 5,000 × 62 when the epoch duration is set to one second. In this paper, we follow Toll et al. (38) to downsample the EEG data to 250 Hz, aiming to reduce the computation cost and improve the inference speed. Similar to (7), we then intercept 120 epochs from each subject's EEG data by a sliding window without overlapping. We set the sliding window with a size of 256, which is about 1 s. The epochs sampled from the data collected with the eyes open and the eyes closed are concatenated in the time dimension. Finally, we copy it for three times in depth dimension. In this way, we have a total of 5,519 epochs, and each epoch $\varepsilon_{i}^{e}$ is a 3 × *C* × 2*T* matrix, where *C* = 62 is the number of channels of EEG data, *T* = 256 denotes the number of time points.

### 4.2. Implementation Details

We randomly select 75% of the subjects. We use their corresponding data clips as our training set, including 3,277 data clips. The remaining data clips serve as our test set, including 2,242 data clips. We train the model for 50 epochs, using a stochastic gradient descent (SGD) optimizer with an initial learning rate of 0.05 and a batch size of 64. All experiments are conducted on a single GTX 1060 GPU.

As for EEG data, we randomly select 75% of the EEG epochs as our training set, containing 4,680 epochs, and the remaining EEG epochs serve as our test set, including 1,560 epochs. We train the model for 70 epochs, using a stochastic gradient descent optimizer with an initial learning rate of 0.005, and with a batch size of 64. All experiments are conducted on a single GTX 1060 GPU.

### 4.3. Comparisons With Other AD Diagnosis Methods

We compare our proposed method with other existing methods. The results is listed in Table 3. Firstly, we compare our proposed attention-based spatial temporal graph convolutional networks with the methods using handcrafted features. We extract the same features as (10) from gait data and feed them into a SVM classifier with the Gaussian (RBF) kernel and a random forest classifier, respectively. The accuracy of the two classifiers are much lower than our proposed attention-based spatial temporal graph convolutional networks (93.09%). These results demonstrate that our proposed attention-based spatial Temporal graph convolutional networks is able to extract more powerful features for the diagnosis of AD.

**Table 3:** Comparison with other methods.

**Methods**	**Data**		**Accuracy**		
	**Gait**	**EEG**	**HC vs. MCI/AD (%)**	**MCI vs. AD (%)**	**Three-way classification (%)**
Handcrafted features + SVM	✓		63.64	57.73	55.45
Handcrafted feature + RF	✓		81.82	57.14	68.18
AST-GCN(ours)	✓		93.09	58.41	68.51
standard CNN		✓	–	69.66	–
EEGnet		✓	–	97.85	–
ResNet 18		✓	–	97.59	–
VGG 13		✓	–	96.48	–
ST-CNN(ours)		✓	–	98.63	–
cascade neural network(ours)	✓	✓	**93.09**	**98.63**	**91.07**

*Standard CNN represents the model we substitute 2D convolution layers with a kernel size of K_s_ × K_t_ for ST-CNN modules. “Handcrafted features + SVM” and “Handcrafted features + RF” indicate the methods using different classifiers with the handcrafted features same as (10). The bold values indicates the best performance that method obtain in that experiment*.

Then we compare the proposed spatial temporal convolutional networks with several baselines on the collected EEG dataset. The baselines include EEGnet (34), ResNet-18 (39), VGG-13 (40), and the standard convolution networks. standard convolutional networks share the same architecture as the spatial temporal convolutional networks but all ST-CNN modules are replaced with 2D convolution layers with a kernel size of *K*_*s*_ × *K*_*t*_. It is observed that our model achieves the best performance on our data set. We believe that the reason is that ST-CNN can extract the spatial and temporal features from EEG data better. Finally, we test our proposed neural network on our test set. The accuracy of binary classification is 93.09%, and the accuracy of the three-way classification is 91.07%. In addition, we introduce a voting mechanism to improve the fault tolerance of the entire framework. We randomly select a subject *s*_*i*_ from the test set and input his gait clip set $G_{i}$ into AST-GCN for classification. If more than 50% of the clips are classified into MCI and AD, all the EEG epochs in $E_{i}$ will be inputted into ST-CNN to perform binary classification of MCI vs. AD. Otherwise, *s*_*i*_ is finally classified into HC. If more than half of the epochs are classified into MCI(AD), then *s*_*i*_ is finally classified into MCI(AD). With the voting mechanism, the framework can achieve an accuracy of 100% on the binary classification of HC vs. MCI/AD, and accuracy of 99.14% on the three-way classification of HC, MCI, and AD.

### 4.4. Ablation Studies

#### 4.4.1. The Effectiveness of the Proposed Component

We conduct experiments on gait data to study the effectiveness of key point filtering and the hourglass attention module. In Table 4, we observe that these two components increase the accuracy from 88.18 to 91.97% and 90.14%, respectively. With both components, we achieve the best performance with an accuracy rate of 93.09%. We believe the reason is that both components can guide the model to focus more on the points more critical to the diagnostic task. Key point filtering removes insignificant points and noise points, and the attention module drives the model to further focus on the important points in key points.

**Table 4:** Ablation study of key point filtering and hourglass attention module on gait data.

**Components**		**Accuracy (%)**
**Key point filtering**	**Hourglass attention module**	
×	×	88.18
✓	×	91.97
×	✓	90.14
✓	✓	93.09

#### 4.4.2. Which Key Points Are Essential for AD Diagnosis?

**Figure 6:**
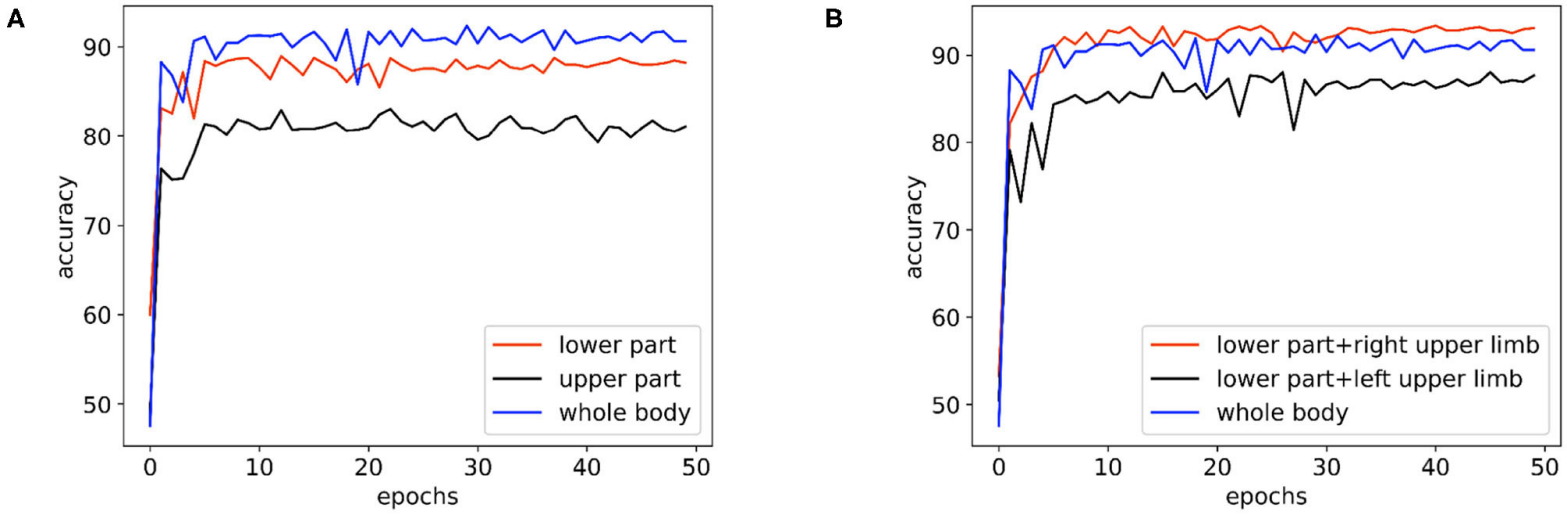
The performance comparison of the basic model on the skeleton sequences composed of different parts: **(A)** The performance of the basic model on the skeleton sequences composed of the lower body, the upper body, and the whole body. **(B)** The performance of the basic model on the datasets of skeleton sequences composed of the whole body, the lower body + the right upper limb, and the lower body + the left upper limb.

In Figure 6A, we compare the performance of the skeleton sequences of the lower body, the upper body, and the whole body. We find that the whole body joint performs best. We consider that this is because all joints can provide more information for diagnosis. In addition, we observe that the lower body joints perform better than upper body joints. We believe the reason is that the behavior of lower body is more relative to early AD diagnosis.

Clinically, it is believed that the left hemisphere of right-handed patients is more sensitive and easier to be affected by AD. As the left hemisphere controls the movement of the right body part, for the right-handed patients, their behaviors of the right body part may provide more information for AD diagnosis. To study this empirically, we further divide the body joints into two more fine-grained groups, namely “lower body + right upper limb” and “lower body + left upper limb.” All subjects in the collected dataset are right-handed. In Figure 6B, “lower body + right upper limb” performs best. these results are consistent with the clinical perspective. Based on such observation, we select the skeleton sequence of “lower body + right upper limb” as a default setting in all experiments.

#### 4.4.3. Where Should We Use the Hourglass Attention Module?

We explore the performance of our model with different placements of the attention module. We try to add the hourglass attention module after the third, sixth, and ninth layer of the basic model, respectively, and add three hourglass attention modules after the 3rd, 6th, and 9th layers. The experimental results are shown in Table 5. We see that using three attention modules additionally includes 67.78% parameters more than using one attention module while decreasing the performance. It is worth nothing that the model with three attention modules outperforms that with one attention module (99.75 vs. 98.04%) in the training phase, but it leads to a worse accuracy (87.97 vs. 90.14%) in the testing phase. We conjecture that adding three attention modules may incur the overfitting issue since a larger network is more likely to lead to overfitting in the case of a limited amount of data (41). We see that adding one attention module after the ninth layer of the basic model achieves the best performance. Therefore, we use the model with an attention module after 9th as the default setting.

**Table 5:** Performance comparison of the models with different hourglass attention module locations.

	**Basic model (%)**	**After 3rd layer (%)**	**After 6th layer (%)**	**After 9th layer**	**After 3rd,6th,9th layers (%)**
Accuracy	88.18	88.76	88.22	**90.14**	87.97

*The bold values indicates the best performance that method obtain in that experiment*.

#### 4.4.4. The Efficiency of Our Method

We conduct an ablation study to validate the effectiveness and efficiency of our method. We replace ST-CNN (classification model with EEG data) in our cascade network with AST-GCN (classification model with gait data). The experimental results are shown in Table 6. Our proposed method with two models significantly outperforms the baseline with one modal (i.e., gait data) while enjoying a faster inference speed (3.99 vs. 7.06 ms) and less parameters (4.72 vs. 9.42M). Since we do not have the EEG data collected from HC regarding the difficulty of collecting them in our experimental environments, we did not compare our method with the EEG-based method, and we leave it for our future work.

**Table 6:** Comparison of the performance and inference speed with different models.

**Cascade stage**		**Accuracy(%)**	**No. of parameters**	**Inference speed (ms)**
**Stage 1**	**Stage 2**			
AST-GCN (gait)	AST-GCN (gait)	74.46	9.42M	7.06
AST-GCN (gait)	ST-CNN (EEG)	**91.07**	4.72M(4.71M+0.01M)	3.99

*The bold values indicates the best performance that method obtain in that experiment*.

## 5. Conclusion

In this paper, we have exploited both the gait and EEG data to achieve a faster and more accurate classification of AD. To this end, we have proposed a cascade neural network. Our proposed neural network consists of two parts. In the first part, we used gait data to distinguish HC from patients. For the purpose of modeling the natural connection among the human joints, we have proposed attention-based spatial temporal graph convolutional networks to extract features to classify the HC and patients. In the second part, we further classify MCI and AD patients with EEG data. Compared with the methods that convert EEG data into the frequency domain, we extract the spatial and temporal features from the original EEG data to distinguish the AD patients from MCI patients. The proposed cascade network has the following advantages: (1) The EEG data from HC are not required in our method, which saves a lot of data collection time. (2) The accuracy of our proposed framework in the three-way classification of HC, MCI, and AD is 91.07%, which is much higher than the method using one modal only (68.18%), and the accuracy in the binary classification of HC vs. MCI/AD reaches 93.09%. It would be interesting to extend this framework to the diagnosis task of other neurological diseases, and we leave it for future work.

## Data Availability Statement

All datasets generated for this study are included in the article/supplementary material.

## Ethics Statement

The studies involving human participants were reviewed and approved by Shenzhen People's Hospital Medical Ethics Committee. The patients/participants provided their written informed consent to participate in this study.

## Author Contributions

All authors listed have made a substantial, direct and intellectual contribution to the work, and approved it for publication.

## Conflict of Interest

The authors declare that the research was conducted in the absence of any commercial or financial relationships that could be construed as a potential conflict of interest.

## References

[1] Brookmeyer R Johnson E Ziegler-Graham K Arrighi HM. Forecasting the global burden of Alzheimer's disease. Alzheimer's Dement. (2007) 3:186–91. 10.1016/j.jalz.2007.04.381 19595937

[2] Patterson CA World Alzheimer Report 2018. London: Alzheimer's Disease International (2018).

[3] Wang WH Hsu YL Pai MC Wang CH Wang CY Lin CW Alzheimer's disease classification based on gait information. In: 2014 International Joint Conference on Neural Networks (IJCNN). Beijing (2014) p. 3251–7. 10.1109/IJCNN.2014.6889762

[4] Varatharajan R Manogaran G Kumar PM Sundarasekar R Wearable sensor devices for early detection of Alzheimer disease using dynamic time warping algorithm. Cluster Comput. (2017) 21:681–90. 10.1007/s10586-017-0977-2

[5] Gao H Liu C Li Y Yang X V2VR: reliable hybrid-network-oriented V2V data transmission and routing considering RSUs and connectivity probability. IEEE Trans Intell Transport Syst. (2020). [Epub ahead of print]. 10.1109/TITS.2020.2983835.

[6] Ieracitano C Mammone N Bramanti A Hussain A Morabito FC A Convolutional Neural Network approach for classification of dementia stages based on 2D-spectral representation of EEG recordings. Neurocomputing. (2019) 323:96–107. 10.1016/j.neucom.2018.09.071

[7] Bi X Wang H. Early Alzheimer's disease diagnosis based on EEG spectral images using deep learning. Neural Netw. (2019) 114:119–35. 10.1016/j.neunet.2019.02.005 30903945

[8] Bennasar M Setchi R Hicks Y Bayer A Cascade classification for diagnosing dementia. In: 2014 IEEE International Conference on Systems, Man, and Cybernetics (SMC). San Diego, CA (2014) p. 2535–40. 10.1109/SMC.2014.6974308

[9] Ning Z Zhang K Wang X Guo L Hu X Huang J Intelligent edge computing in internet of vehicles: a joint computation offloading and caching solution. IEEE Trans Intell Transport Syst. (2020). [Epub ahead of print]. 10.1109/TITS.2020.2997832.

[10] Seifallahi M Soltanizadeh H Mehraban AH Khamseh F Alzheimer's disease detection using skeleton data recorded with Kinect camera. Cluster Comput. (2019) 23:1469–81. 10.1007/s10586-019-03014-z

[11] Yu Y Liu S Guo L Yeoh PL Vucetic B Li Y CrowdR-FBC: a distributed fog-blockchains for mobile crowdsourcing reputation management. IEEE Intern Things J. (2020) 7:8722–35. 10.1109/JIOT.2020.2996229

[12] Anderer P Saletu B Klöppel B Semlitsch HV Werner H Discrimination between demented patients and normals based on topographic EEG slow wave activity: comparison between z statistics, discriminant analysis and artificial neural network classifiers. Electroencephalogr Clin Neurophysiol. (1994) 91:108–17. 10.1016/0013-4694(94)90032-9 7519140

[13] Pritchard WS Duke DW Coburn KL Moore NC Tucker KA Jann MW. EEG-based, neural-net predictive classification of Alzheimer's disease versus control subjects is augmented by non-linear EEG measures. Electroencephalogr Clin Neurophysiol. (1994) 91:118–30. 10.1016/0013-4694(94)90033-7 7519141

[14] Ning Z Dong P Wang X Hu X Guo L Hu B Mobile edge computing enabled 5G health monitoring for internet of medical things: a decentralized game theoretic approach. IEEE J Select Areas Commun. (2020). [Epub ahead of print].

[15] Trambaiolli LR Lorena AC Fraga FJ Kanda PAM Anghinah R Nitrini R. Improving Alzheimer's disease diagnosis with machine learning techniques. Clin EEG Neurosci. (2011) 42:160–5. 10.1177/155005941104200304 21870467

[16] Rossini PM Buscema M Capriotti M Grossi E Babiloni C. Is it possible to automatically distinguish resting EEG data of normal elderly vs. mild cognitive impairment subjects with high degree of accuracy? Clin Neurophysiol. (2008) 119:1534–45. 10.1016/j.clinph.2008.03.026 18485814

[17] Gao H Xu Y Yin Y Zhang W Li R Wang X Context-aware QoS prediction with neural collaborative filtering for internet-of-things services. IEEE Intern Things J. (2020) 7:4532–42. 10.1109/JIOT.2019.2956827

[18] Callisaya ML Launay CP Srikanth V Verghese J Allali G Beauchet O. Cognitive status, fast walking speed and walking speed reserve-the Gait and Alzheimer Interactions Tracking (GAIT) study. GeroScience. (2017) 39:231–9. 10.1007/s11357-017-9973-y 28374167 PMC5411364

[19] Ning Z Zhang K Wang X Obaidat MS Guo L Hu X Joint computing and caching in 5G-envisioned internet of vehicles: a deep reinforcement learning-based traffic control system. IEEE Trans Intell Transport Syst. (2020). [Epub ahead of print]. 10.1109/TITS.2020.2970276.

[20] Beauchet O Launay CP Sekhon H Montembeault M Allali G. Association of hippocampal volume with gait variability in pre-dementia and dementia stages of Alzheimer disease: results from a cross-sectional study. Exp Gerontol. (2019) 115:55–61. 10.1016/j.exger.2018.11.010 30447261

[21] Elbaz A Artaud F Singh-Manoux A Dumurgier J. Gait speed and decline in gait speed as predictors of incident dementia. Innov Aging. (2017) 1:75. 10.1093/geroni/igx004.310 27302701

[22] Ardle RM Morris R Wilson JB Galna B Thomas AJ Rochester LR. What can quantitative gait analysis tell us about dementia and its subtypes? A structured review. J Alzheimer's Dis. (2017) 60:1295–312. 10.3233/JAD-170541 29036826

[23] Morris R Lord S Lawson RA Coleman S Galna B Duncan GW. Gait rather than cognition predicts decline in specific cognitive domains in early Parkinson's disease. J Gerontol Ser A. (2017) 72:1656–62. 10.1093/gerona/glx071 28472409 PMC5861960

[24] Hsu YL Chung PC Wang WH Pai MC Wang CY Lin CW. Gait and balance analysis for patients with Alzheimer's disease using an inertial-sensor-based wearable instrument. IEEE J Biomed Health Informatics. (2014) 18:1822–30. 10.1109/JBHI.2014.2325413 25375679

[25] Gao H Kuang L Yin Y Guo B Dou K Mining consuming behaviors with temporal evolution for personalized recommendation in mobile marketing apps. Mobile Netw Appl. (2020) 25:1233–48. 10.1007/s11036-020-01535-1

[26] Wang X Ning Z Guo S Multi-agent imitation learning for pervasive edge computing: a decentralized computation offloading algorithm. IEEE Trans Parallel Distrib Syst. (2020) 32:411–25. 10.1109/TPDS.2020.3023936

[27] Yu Y Liu S Yeoh P Vucetic B Li Y LayerChain: a hierarchical edge-cloud blockchain for large-scale low-delay IIoT applications. IEEE Trans Indus Informatics. (2020) [Epub ahead of print]. 10.1109/TII.2020.3016025.

[28] Yan JH Rountree SD Massman PJ Doody R Li H. Alzheimer's disease and mild cognitive impairment deteriorate fine movement control. J Psychiatr Res. (2008) 42:1203–12. 10.1016/j.jpsychires.2008.01.006 18280503

[29] Choi JS Oh HS Kang DW Mun KR Choi MH Lee SJ Comparison of gait and cognitive function among the elderly with Alzheimer's disease, mild cognitive impairment and healthy. Int J Precis Eng Manufact. (2011) 12:169–73. 10.1007/s12541-011-0024-9

[30] Bashivan P Rish I Yeasin M Codella N Learning representations from EEG with deep recurrent-convolutional neural networks. CoRR. (2016) abs/1511.06448.

[31] Yan S Xiong Y Lin D. Spatial temporal graph convolutional networks for skeleton-based action recognition. In: AAAI. New Orleans, LA (2018).

[32] Wang X Ning Z Guo S Wang L Imitation learning enabled task scheduling for online vehicular edge computing. IEEE Trans Mobile Comput. (2020). [Epub ahead of print]. 10.1109/TMC.2020.3012509.

[33] Yang Z Li Y Yang J Luo J Action recognition with spatio-temporal visual attention on skeleton image sequences. IEEE Trans Circuits Syst Video Technol. (2019) 29:2405–15. 10.1109/TCSVT.2018.2864148

[34] Lawhern V Solon AJ Waytowich NR Gordon SM Hung CP Lance B. EEGNet: a compact convolutional network for EEG-based brain-computer interfaces. J Neural Eng. (2018) 15:056013. 10.1088/1741-2552/aace8c 29932424

[35] Chattopadhyay P Sural S Mukherjee J Frontal gait recognition from occluded scenes. Pattern Recogn Lett. (2015) 63:9–15. 10.1016/j.patrec.2015.06.004

[36] Fang J Wang T Li C Hu X Ngai ECH Seet BC Depression prevalence in postgraduate students and its association with gait abnormality. IEEE Access. (2019) 7:174425–37. 10.1109/ACCESS.2019.2957179

[37] Beyrami SMG Ghaderyan P A robust, cost-effective and non-invasive computer-aided method for diagnosis three types of neurodegenerative diseases with gait signal analysis. Measurement. (2020) 156:107579 10.1016/j.measurement.2020.107579

[38] Toll RT Wu W Naparstek S Zhang Y Narayan M Patenaude B. An electroencephalography connectomic profile of posttraumatic stress disorder. Am J Psychiatry. (2020) 177:233–43. 10.1176/appi.ajp.2019.18080911 31964161

[39] He K Zhang X Ren S Sun J Deep residual learning for image recognition. In: 2016 IEEE Conference on Computer Vision and Pattern Recognition (CVPR). Las Vegas, NV (2016) p. 770–8. 10.1109/CVPR.2016.90

[40] Simonyan K Zisserman A Very deep convolutional networks for large-scale image recognition. CoRR. (2015) abs/1409.1556.

[41] Srivastava N Hinton GE Krizhevsky A Sutskever I Salakhutdinov R Dropout: a simple way to prevent neural networks from overfitting. J Mach Learn Res. (2014) 15:1929–58.

